# Thermal–Mechanical Coupling Behavior of Directional Polymethylmethacrylate under Tension and Compression

**DOI:** 10.3390/polym10111279

**Published:** 2018-11-16

**Authors:** Hui Guo, Chunjiang Lu, Yu Chen, Junlin Tao, Longyang Chen

**Affiliations:** 1 School of Civil Engineering and Architecture, Southwest University of Science and Technology, Mianyang 621010, China; guohui56789@126.com (H.G.); ghlwtg@126.com (C.L.); chenyu456789@126.com (Y.C.); 2 Shock and Vibration of Engineering Materials and Structures Key Laboratory of Sichuan Province, Southwest University of Science and Technology, Mianyang 621010, China; 3 School of Aeronautics, Northwestern Polytechnical University, Xi’an 710072, China; yang218cly@aliyun.com

**Keywords:** directional polymethylmethacrylate, tensile and compressive behavior, temperature effect, rate-dependence, constitutive modeling

## Abstract

In this work, the quasi-static and dynamic mechanical behavior of directional polymethylmethacrylate is investigated under conditions of uniaxial compression and tension. The main purpose of this investigation is to discuss the effect of strain rate and temperature on the deformation characteristics and failure of such material. Research was carried out with the use of an electric universal testing machine and split Hopkinson bars, which were equipped with high- and low-temperature control systems. The experimental methods for studying the tensile and compressive response of polymer materials under different testing conditions were validated by one-dimensional stress wave theory and digital-image correlation technique. The finite deformation stress–strain behaviors of the samples under different loading condition were obtained with a constant temperature ranging from 218 to 373 K. The experimental results showed that the uniaxial tensile and compressive behaviors of directional polymethylmethacrylate under finite deformation are strongly dependent on temperature, decreased tensile and compressive stress of the material under different strain levels, and increased temperature. Meanwhile, the dynamic tensile and compressive stresses of the material are much higher than the quasi-static stresses, showing the strain-rate strengthening effect. Moreover, the tensile and compressive mechanical behavior of directional polymethylmethacrylate has significant asymmetry. Finally, a visco-hyperelastic model is established to predict the rate-dependence mechanical behavior of directional polymethylmethacrylate at different temperatures.

## 1. Introduction

Directional polymethylmethacrylate, with good transmittance, light mass, high strength, and aging resistance, is widely used in the field of aviation, such as in aircraft canopies, windshields, observation windows, and other transparent parts. In recent years, failure accidents of directional polymethylmethacrylate in aircraft components have frequently occurred, and the development of new high-performance aircraft has also presented stronger requirements for its mechanical properties. Therefore, the mechanical behavior of directional polymethylmethacrylate has attracted increasing attention from researchers. If an aircraft encounters a bird during flight, it can cause directional polymethylmethacrylate as windshield material to deform at a high strain rate. Moreover, the material is affected by temperature variations between ground and air, and the stresses generated by thermal expansion and contraction have great influence on the reliability of the windshield. It is therefore necessary to study the mechanical properties of directional polymethylmethacrylate under extreme environments, such as high temperature and high strain rate.

More recently, scholars have conducted research on the mechanical properties of directional polymethylmethacrylate under dynamic load. For example, Li et al. [1] carried out quasi-static and dynamic compressive experiments under isothermal conditions and found that with increased strain rate, the compressive yield strength increased and the mechanical properties changed from toughness to brittleness. Chen et al. [2] studied the uniaxial tensile and compressive mechanical properties of polymethylmethacrylate using split Hopkinson bar systems, and determined that the stress–strain behavior of the material under dynamic tension was different from that under dynamic compression. More recently, Forquin et al. [3] studied the confined behavior of polymethylmethacrylate at strain rates ranging from 10^−3^/s to 10^3^/s, and their results showed that yield strength was greatly affected by strain rate, but less affected by confining pressure. Jin et al. [4] studied the failure behavior of polymethylmethacrylate under quasi-static and dynamic loading conditions, and analyzed the compression and shear deformation characteristics of the material at different strain rates. Jali et al. [5] carried out a dynamic impact test on polymethylmethacrylate, and obtained its fracture mechanical properties and failure mechanism under impact load. Experimental studies have also been conducted to investigate the thermodynamic properties of polymethylmethacrylate at different temperatures. In earlier research, Marshall et al. [6] studied its fracture toughness in a wide range of temperatures, and concluded that the change in fracture toughness can be reflected by modulus change with temperature and rate. Blumenthal et al. [7] conducted uniaxial compression stress–strain measurements on polymethylmethacrylate at different temperatures, and found that its strength was sensitive to temperature. Subsequently, Palm et al. [8] studied its nonlinear mechanical properties near the glass transition temperature, and established a three-dimensional constitutive relation suitable for describing the stress–strain response. Abdel-Wahab et al. [9] studied the temperature-dependent mechanical properties of polymethylmethacrylate under three-point bending and uniaxial tension-loading conditions, and obtained a variation of failure behavior with temperature. Liu et al. [10] studied its thermodynamic behavior in a certain temperature range, and established a physical constitutive relation. In addition, some scholars have put forward suitable constitutive models for polymethylmethacrylate materials through experimental research and theoretical analysis. For example, Menikoff et al. [11] produced a three-dimensional elastoplastic constitutive model suitable for polymethylmethacrylate at high pressure based on experimental data of rate-dependent mechanical behavior. Dar et al. [12] also developed a nonlinear viscoelastic model to describe the rate-dependent compressive behavior of polymethylmethacrylate, and validated the applicability of the constitutive model by quasi-static and dynamic experimental data. In summary, many scholars have carried out preliminary studies on the mechanical behavior and failure characteristics of polymethylmethacrylate at different loading conditions, and have obtained valuable experimental results. However, there remain many questions regarding the tensile and compressive mechanical properties under the coupled effect of temperature and dynamic loading. Therefore, a comprehensive study on the quasi-static and dynamic tensile and compressive mechanical response of directional polymethylmethacrylate with particular focus on the influence of temperature is still needed.

The main purpose of this paper is to examine the quasi-static and dynamic tensile and compressive mechanical response of a new kind of directional polymethylmethacrylate at different temperatures. For this reason, a systematic experimental study on the nonlinear mechanical behavior of the material under the coupled effects of temperature and strain rate was carried out by a corresponding improved experimental apparatus. The stress–strain characteristics of directional polymethylmethacrylate under uniaxial tensile and compressive loadings are analyzed and discussed in this paper. Based on the results and theoretical analysis, a three-dimensional visco-hyperelastic model is proposed to describe the temperature-dependent mechanical behavior of directional polymethylmethacrylate under different loading conditions. Finally, the applicability of the resulting model is verified by the experimental results of the material.

## 2. Experimental Protocol

### 2.1. Material and Specimens

**Figure 1:**
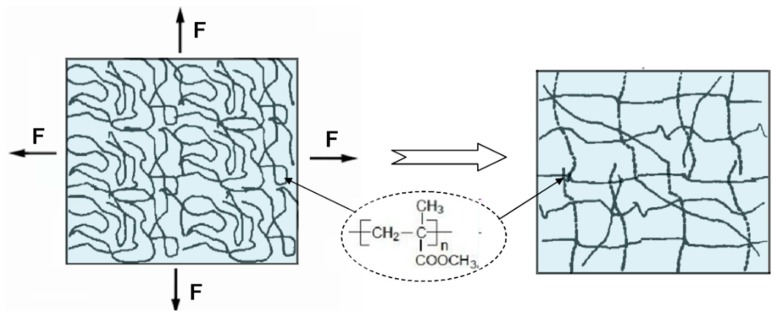
Comparison of molecular chains of nondirectional polymethylmethacrylate and directional polymethylmethacrylate after processing.

The material studied in this paper is a new type of polymethylmethacrylate, which is referred to as directional polymethylmethacrylate. The test material was provided by the Beijing Institute of Aeronautical Materials. Directional polymethylmethacrylate was obtained by the biaxial hot-drawn, cooling, and curing process of polymethylmethacrylate in the vicinity of the glass transition temperature. As Figure 1 shows, the molecular chain of polymethylmethacrylate was changed from coil conformation to extended conformation through the above treatment process, which causes a decrease in conformational entropy. The obtained material has excellent optical and thermodynamic stability, and also has the anisotropy of mechanical, optical, and thermal properties.

**Figure 2:**
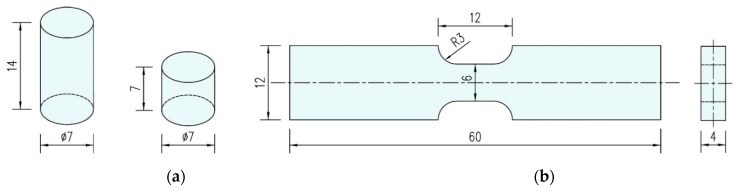
Geometric size of test piece (in mm): (**a**) compression specimens, (**b**)tension specimen.

According to the national standard “Fiber-reinforced plastics composites—determination of compressive properties” [13], the directional polymethylmethacrylate specimens used in the quasi-static compression experiment was designed as a cylinder with dimensions of ⌀ 7 × 14 mm. In order to meet the requirements of experimental precision, the surface roughness of the specimens was less than 0.01 mm, and the nonparallelism of the upper and lower end faces was less than 0.1% of the specimen height. A suitable sample size could reduce errors due to radial and longitudinal inertia as well as friction effects in split Hopkinson pressure bar tests. Therefore, the dimension of directional polymethylmethacrylate specimens used in dynamic compression experiments was selected as a ⌀ 7 × 7 mm cylinder on the basis of the ASM Metals Handbook [14]. For the quasi-static and dynamic tensile tests, the specimens were designed as flat plates with a double arc transition form, as shown in Figure 2. The arc transition zone ensures the specimen fracture in the effective section, and effectively reduces the phenomenon of stress concentration on the specimen.

### 2.2. Quasi-Static Testing Method

**Figure 3:**
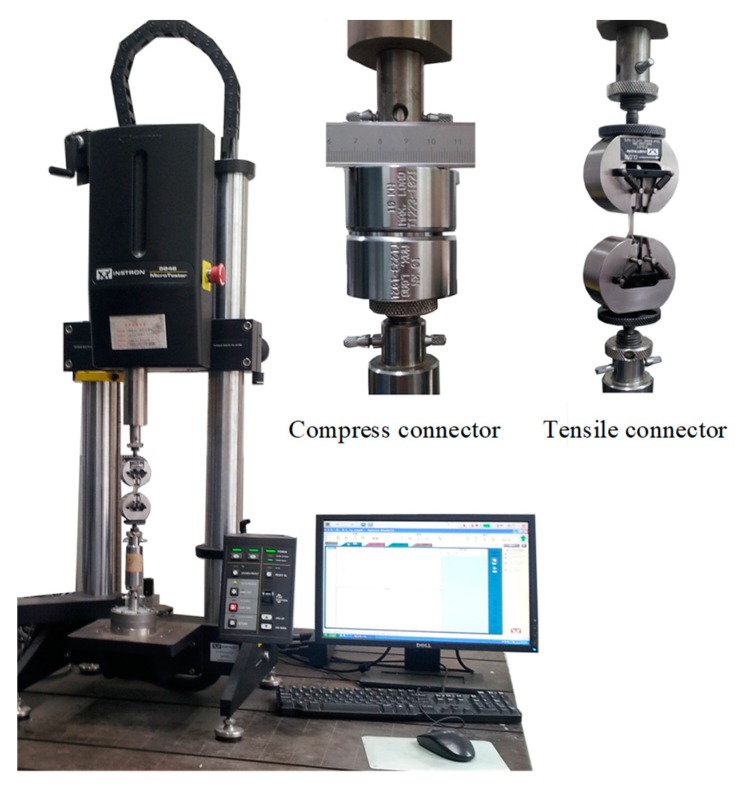
Schematic diagram of testing machine and compress/tensile connector.

Quasi-static tensile and compressive tests under various conditions were conducted with an electric universal testing machine. This machine can realize uniaxial tensile and compressive testing at a low strain rate range of 0.001–0.1 s by replacing the compress/tensile connector. A schematic diagram of the testing machine and the compress/tensile connector is shown in Figure 3. During the tests, the load was measured by a load sensor installed at the bottom of the loading rod, while displacement was automatically recorded by the test machine according to the movement distance of the moving loading bar. The accuracy of the tension and compression dual-purpose load sensor was 0.5%, and the measuring range was 2–100% of the sensor capacity. According to the data recorded by the tester, the engineering stress of directional polymethylmethacrylate can be calculated by the load divided by the initial cross-sectional area of the specimen, and the engineering strain can be obtained by the displacement divided by the initial length of the specimen. Based on the assumption of volume invariance of the material during deformation, the true stress and strain of test specimens under quasi-static loading are obtained with the mathematical calculation method. In addition, the strain rates in quasi-static testing are calculated by the following formula:

(1) $$\dot{\varepsilon }=\frac{d\varepsilon }{dt}=\frac{\Delta l}{l_{0}\cdot \Delta t}=\frac{v}{l_{0}}\;$$

where Δ*l* is elongation of the specimen after deformation, Δ*t* is the deformation time, *l*_0_ is the initial length of the specimen, and *ν* is the loading velocity controlled by the mechanical sensor.

### 2.3. Dynamic Testing Method

**Figure 4:**
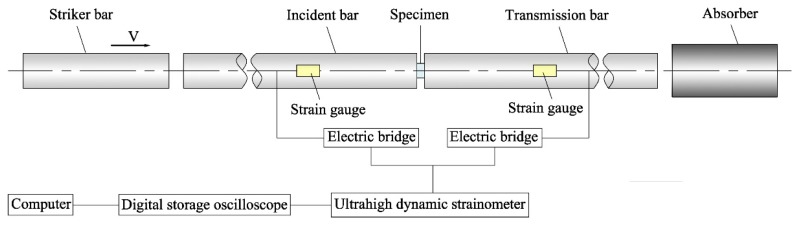
Schematic diagram of the split Hopkinson pressure bar apparatus.

**Figure 5:**
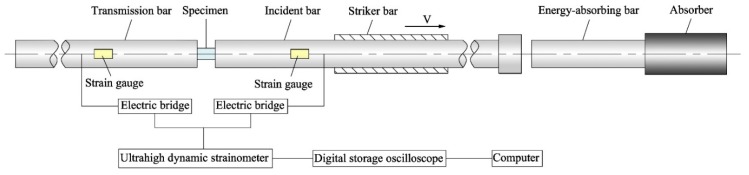
Schematic diagram of the split Hopkinson tensile bar apparatus.

Dynamic tensile and compressive tests of directional polymethylmethacrylate under various conditions were carried out on the split Hopkinson pressure bar (SHPB) and the split Hopkinson tensile bar (SHTB), respectively. This split Hopkinson bar apparatus can achieve uniaxial tensile and compressive testing for a high strain rate range of 10^2^–10^4^/s. Schematic diagrams of SHPB and SHTB used in this study are shown in Figure 4 and Figure 5. The split Hopkinson bar apparatus is mainly composed of three parts: striker, incident, and transmission bars. In SHPB experiments, the striker bar is driven by high-pressure gas to impact the end of the incident bar at a certain velocity, thereby producing an elastic stress wave in the incident bar. Once the elastic wave propagating along the incident bar reaches the bar/specimen interface, a portion of the wave pulse is reflected to form a reflected wave. When the remaining wave pulse passes through the specimen and enters the transmission bar, it forms a transmitted wave. The duration and amplitude of the elastic wave through the incident and transmission bars are recorded by strain gauges on the bars. The wave pulse measured by the strain gauges is a function of time, and it can be used to determine the force and strain rate on the bar/specimen interface. When the specimen is uniformly deformed, the stress of the specimen can be calculated by the force divided by the cross-sectional area of the specimen, while the strain can be obtained by integrating the strain rate accordingly to get the stress–strain curves of the specimen under dynamic loading. Based on the assumption of one-dimensional stress wave, the strain rate, strain, and stress histories of directional polymethylmethacrylate under dynamic loading can be given as follows:

(2) $$\dot{\varepsilon }\left(t\right)=\frac{C_{b}}{l}(\varepsilon_{i}-\varepsilon_{r}-\varepsilon_{t})\;$$

(3) $$\varepsilon \left(t\right)=\frac{C_{b}}{l}\int_{0}^{t}\left(\varepsilon_{i}-\varepsilon_{r}-\varepsilon_{t}\right)d\tau \;$$

(4) $$\sigma \left(t\right)=\frac{E_{b}A_{b}}{2A}(\varepsilon_{i}+\varepsilon_{r}+\varepsilon_{t})\;$$

where *A*_b_, *C*_b_, and *E*_b_ are the cross-sectional area, stress–wave velocity, and elastic modulus of the incident/transmission bars, respectively; *l* and *A* are the initial length and cross-sectional area of the samples, respectively; and *ε_i_*, *ε_r_*, and *ε_t_* are the incident, reflected, and transmission wave pulses in the incident/transmission bars, respectively. The principle and data analysis of the SHTB experiment are similar to those of the SHPB experiment discussed above. The main differences lie in the generation schemes of the tensile stress pulse, the specimen geometry, and the method of connecting the specimen to the incident and transmission bars. Here, an adhesive connection is used between the specimen and the elastic bars in the SHTB experiments. After the epoxy adhesive is cured for 24 h, the bonding strength of the joints reaches the maximum and is sufficient to withstand the tensile force of the joint in the test without damage.

**Figure 6:**
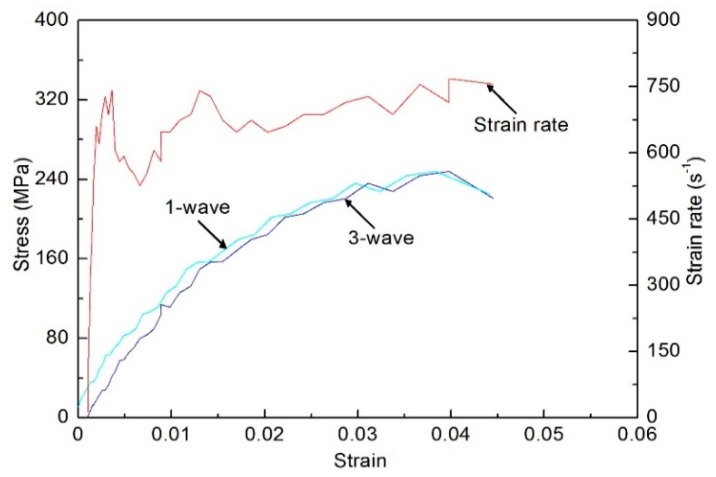
Analysis of stress uniformity for a specimen with a temperature of 288 K under dynamic compression.

Recent studies have shown that inertia and wave-propagation effects within the test specimen can significantly affect stress distribution along the length of the specimen in SHPB and SHTB tests [14,15]. Meanwhile, the slow-wave velocity in the polymer material makes it difficult to reach longitudinal stress equilibrium during the tests. Therefore, in order to ensure the reliability of the experimental data, it is necessary to analyze the stress equilibrium of the specimen during dynamic loading. Previous studies showed that stress equilibrium can be achieved by making the width of the incident pulse sufficiently longer than the stress-wave time passing through the specimen [15,16]. More specifically, three to four reflections of stress wave through the specimen should be demanded before stress equilibrium is achieved in the entire specimen. Therefore, a triangular incident wave containing a slow rising stage is needed to ensure that adequate wave reflections occur within the entire specimen. The triangular incident wave can be achieved by adding a thin copper sheet at the impact end of the incident bar. With the impact of the striker bar, the plastic deformation of the copper sheet causes a triangular wave pulse signal to appear in the incident bar. The rise time of the triangular wave can be adjusted by the geometric size of the copper sheet. Through trial and error, the optimal geometric dimension of the copper sheet was chosen as 0.3 mm in thickness and 3–5 mm in diameter for different conditions. In addition, the copper sheet as a pulse shaper can filter out high-frequency signals, thus reducing the effect of waveform dispersion. It can also produce a flat reflection wave, which is beneficial to realize a loading process under constant strain rate, as shown in Figure 6. Once the specimens satisfy the assumption of stress uniformity in the SHPB and SHTB experiments, the sum of the strain increments of the incident and reflected waves in the incident bar is equal to the strain increment of the transmitted wave in the transmission bar. Thus Equations (2)–(4) can be simplified as follows:

(5) $$\dot{\varepsilon }\left(t\right)=\frac{C_{b}}{l}(\varepsilon_{i}-\varepsilon_{r}-\varepsilon_{t})=-\frac{2C_{b}\varepsilon_{r}}{l}\;$$

(6) $$\varepsilon \left(t\right)=\frac{C_{b}}{l}\int_{0}^{t}\left(\varepsilon_{i}-\varepsilon_{r}-\varepsilon_{t}\right)d\tau =-\frac{2C_{b}}{l}\int_{0}^{t}\varepsilon_{r}d\tau \;$$

(7) $$\sigma \left(t\right)=\frac{E_{b}A_{b}}{2A}(\varepsilon_{i}+\varepsilon_{r}+\varepsilon_{t})=\frac{E_{b}A_{b}\varepsilon_{t}}{A}\;$$

The method of determining the dynamic stress–strain curves of the specimens by Equations (5)–(7) is termed a one-wave analysis, while the method of the dynamic stress–strain curves determined by Equations (2)–(4) is termed a three-wave analysis. Whether the specimen reaches stress equilibrium can be checked by comparing the one-wave and three-wave stress–strain responses [14,17]. The comparison of one-wave and three-wave stress–strain responses of the directional polymethylmethacrylate specimen under dynamic compression is shown in Figure 6. The results show that the compressive stress–strain curve obtained by the two analysis methods is essentially comparable except for the initial section, which indicates that the assumption of stress uniformity can be satisfied in the SHPB experiments. Meanwhile, after stress uniformity is reached, an approximate constant strain rate is achieved within almost the entire deformation process.

In order to analyze the deformation uniformity of the specimen under dynamic tension, the deformation field is quantitatively measured by high-speed photography and digital image correlation. Digital image correlation (DIC), also known as digital speckle correlation, is based on the principle that the displacement vector of the pixel is obtained by tracking (or matching) the position of the same pixel in two speckle images before and after specimen deformation. The use of the digital-image correlation method for displacement and deformation measurements needs three steps: specimen preparation, image acquisition before and after loading, and digital-image correlation calculation. In the DIC method, since the deformation information of the specimen needs to be obtained by processing the digital image of the specimen surface, the imaging surface of the specimen must have a random grayscale distribution (i.e., random speckle pattern), which changes with the deformation of the specimen surface. At present, the main method of obtaining a random speckle pattern is to manually spray black and white paint on the surface of the specimen. During the measurement process, the speckle pattern is detected by a charge-coupled device (CCD) camera and stored in a computer. Then, the deformation information on the surface of the specimen is obtained by a DIC algorithmic program. The algorithmic program calculates the displacement vector of each pixel by comparing the digital images of the surface of the tested specimen in different states. The digital speckle image before deformation is usually referred to as a reference image, and the image after deformation is called the deformed image. The algorithmic calculation process is as follows: First, it takes the measured point as the center to select a square subimage from the reference image, which is called the reference subset. Meanwhile, a square subimage is also selected at the corresponding pixel points in the deformed image, which is called the search subset. A subset of the same size as the reference subset is selected in the search subset, which is called the deformed subset. Then, the corresponding relationship between each deformed subset and the reference subset is quantified according to the predefined correlation function, and a correlation coefficient distribution map is obtained. The position of the extreme point of the maximum or minimum correlation coefficient corresponds to the position of the target subset, and the difference between the coordinates of the center point of the target subset and the center point of the reference subset is the displacement vector of the measured point. Using the same method, full-field displacement of the specimen surface can be obtained by the above-mentioned operation on each pixel of the speckle image before and after deformation. Finally, considering the curvature of the specimen surface, the strain of each point on the surface can be obtained by calculating the parameters of affine deformation and the gradient of deformation, thus the strain field can be obtained. It can be seen from this acquisition process that accurate measurement of the displacement field is key to the DIC method, because strain measurement is based on displacement data. Pan et al. discussed the accuracy of the displacement measurement of the DIC method and found that its value is an unbiased estimation of the true value in the absence of interpolation errors, that is, the displacement measurement result is accurate [18]. However, in practice, interpolation error is unavoidable; the interpolation method directly affects the accuracy of the final calculation, convergence characteristics, and calculation efficiency. Therefore, the choice of the interpolation method is key in the DIC method. At present, commonly used methods have polynomial, spline, and B-spline interpolations. Schreier et al. studied the choice of grayscale interpolation algorithm and found that the quintic B-spline interpolation was the best one [19]. Based on their research, we chose the quintic B-spline interpolation in the algorithm program, which can completely meet the accuracy requirements.

**Figure 7:**
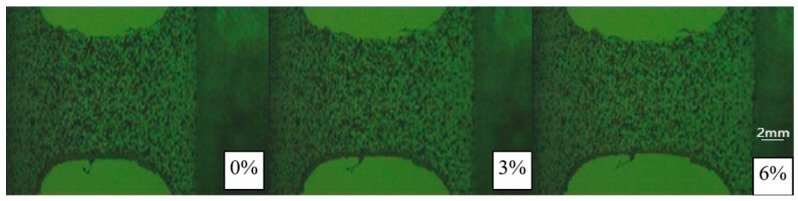
Deformation diagram of specimen corresponding to different strains at a temperature of 255 K.

**Figure 8:**
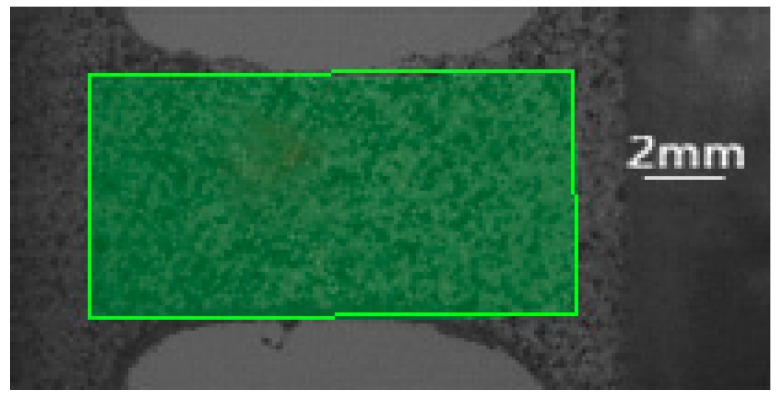
Reading image area of digital-image correlation (DIC) calculation.

**Figure 9:**
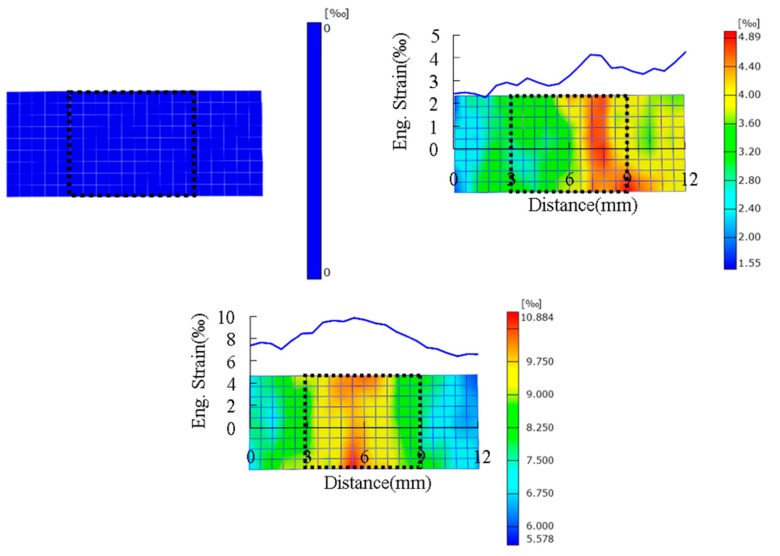
Strain fields under different deformation states obtained by the DIC technique.

A deformation diagram of the specimen corresponding to different strains at a temperature of 255 K is shown in Figure 7. The corresponding engineering strain is given in the bottom-right corner, and its value is calculated by dividing the displacement of the two loading ends by the length of the specimen. It can be seen from Figure 7 that the deformations of the left and right sides of the specimen are basically symmetrical in the whole deformation process, and there is no obvious necking phenomenon, which also shows that the stress on the left and right ends of the specimen is basically uniform. The quantitative strain field can be obtained by processing the deformation diagram with the DIC technique. The reading image area in Figure 8 and Figure 9 shows the strain fields under different deformation states calculated by the DIC technique. It can be found that in the initial deformation stage, the strain near the end of the incident bar (right end) is slightly larger than the strain near the end of the transmission bar, but deformation is approximately uniform when the strain in the gauge section is about 8‰. Meanwhile, the strain in the gauge section of the specimen is larger than the strain outside the gauge section after deformation is uniform.

### 2.4. Temperature-Control Technology

**Figure 10:**
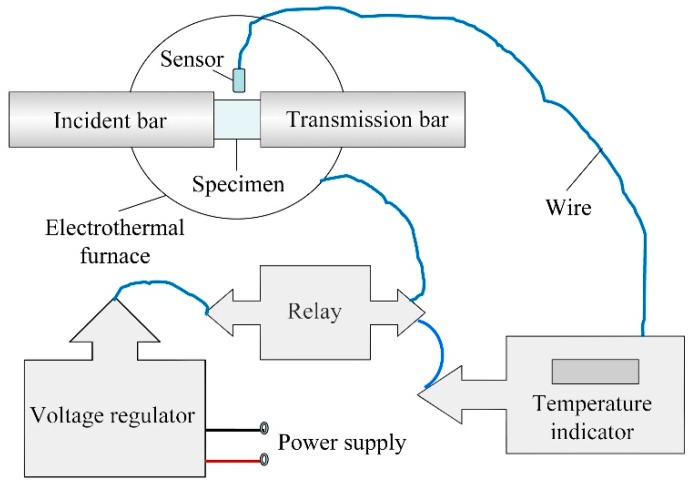
Principle diagram of high-temperature control system.

**Figure 11:**
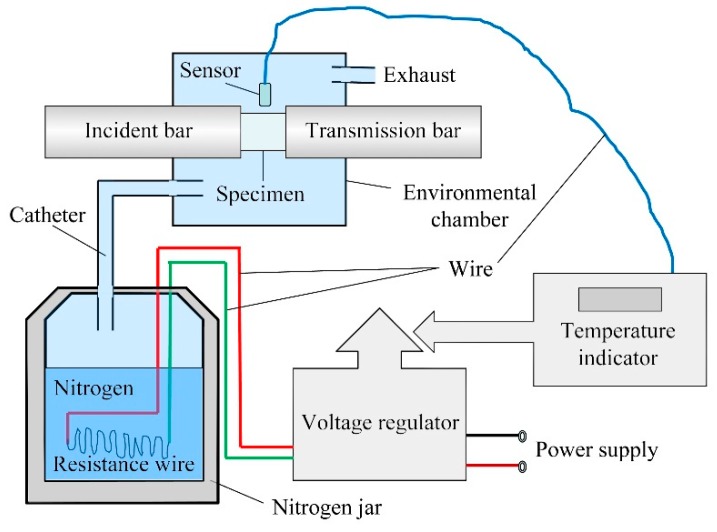
Principle diagram of low-temperature control system.

The application environment of directional polymethylmethacrylate in aeronautical structures determines the necessity of studying the influence of temperature on its mechanical properties. The mechanical properties of the material have an obvious temperature effect; the temperature-dependence effect is more obvious especially in the vicinity of the glass transition temperature. Up to now, the influence of temperature on the mechanical behavior of polymers has not been extensively studied under different strain rates [7,8]. Therefore, two sets of simple high- and low-temperature control systems were designed to study the mechanical behavior of directional polymethylmethacrylate at different temperatures, which can achieve mechanical testing in a temperature range from 218 to 373 K. The high- and low-temperature environmental control systems are shown in Figure 10 and Figure 11. The test piece is heated by an electrothermal furnace in the high-temperature control system, while it is cooled by liquid nitrogen in the low-temperature control system. During the heating and cooling process, a thermocouple that is slightly touched with the outer surface of the specimen is used to feed back and control the specimen temperature. After the temperature reaches a predetermined value, the specimen temperature maintains a steady state for at least 5 min before the test is started.

## 3. Experimental Results and Analysis

The quasi-static and dynamic tensile and compressive mechanical properties of directional polymethylmethacrylate under different temperatures were investigated by the above testing technology. The test results and a discussion are provided in the following sections.

### 3.1. Quasi-Static and Dynamic Compressive Response at Different Temperatures

**Figure 12:**
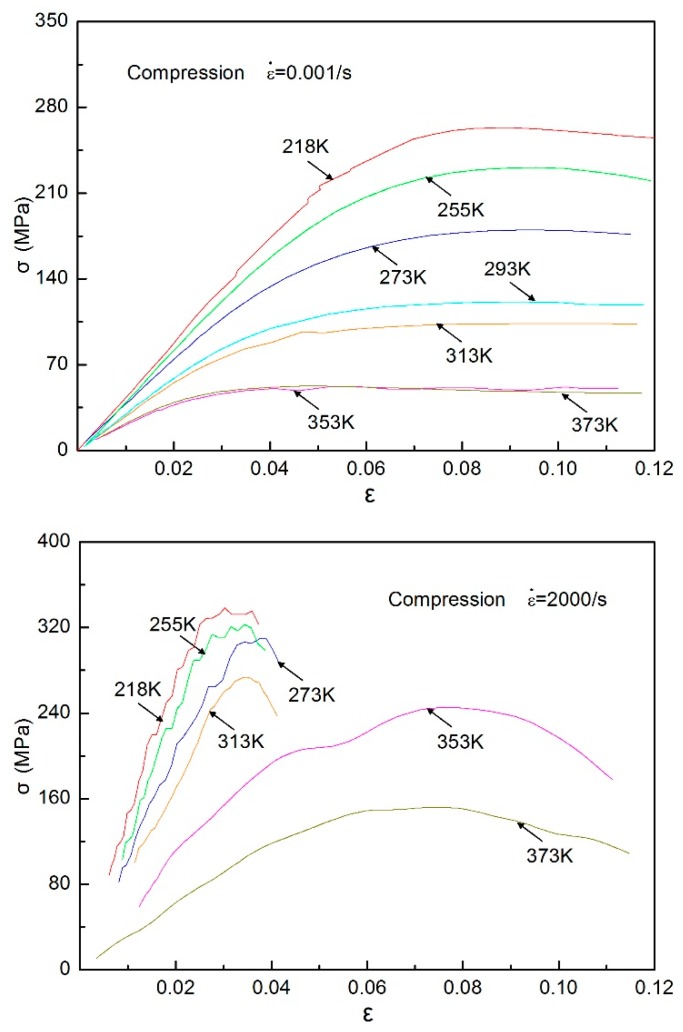
Quasi-static and dynamic compressive stress–strain curves of directional polymethylmethacrylate at different temperatures.

The quasi-static and dynamic compressive experiments for directional polymethylmethacrylate specimens were carried out under different temperatures. The compressive stress–strain curves of the specimens are given over a wide temperature range from 218 to 373 K, as shown in Figure 12. It can be seen that the peak stress, initial elastic modulus, and strain-hardening rate of the material decrease with the increase of temperature. For the low strain rate of 0.001/s, the curves of 353 and 373 K are almost coincident with each other, which shows that the material softened when the temperature reached or approached the glass transition temperature of directional polymethylmethacrylate, and the stress was already very small. For the high strain rate of 2000/s, the invalid experimental data in this area were omitted because the required constant strain rate was not reached in the initial segment of the stress–strain curve. It can be found from the dynamic stress–strain curves that when the test temperature increased from 313 to 373 K, the dynamic modulus of directional polymethylmethacrylate significantly decreased. The dynamic modulus decreased slightly when the temperature rose from 218 to 313 K. In addition, when the test temperature was less than or equal to 313 K, the material underwent fracture failure without apparent plastic flow. When the test temperature rose to 353 K, the stress–strain curves showed highly nonlinear behavior, and the specimen underwent great plastic flow deformation before failure occurred. Meanwhile, there was a slight decrease in the flow stress before the specimen failed; that is, the maximum stress in the process of testing was not the stress at which the specimen failed. A similar phenomenon was also found in the research conclusions of polyamide and polyetheretherketone [20]. This may be because movement of molecular segments at higher temperatures is relatively easy; when stress increases to a certain value, partial molecular segments are destroyed and lose carrying capacity, but most molecular chains can still withstand the load until the specimen fails.

**Figure 13:**
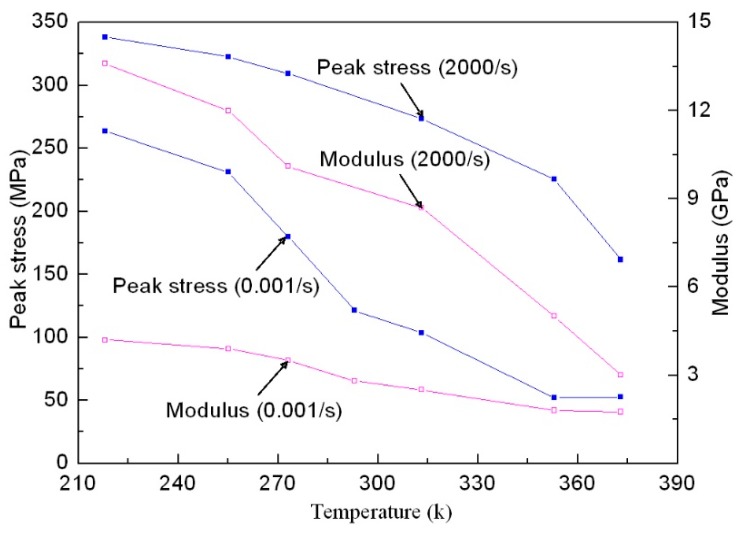
Relation of the peak stress and modulus of directional polymethylmethacrylate with temperature under quasi-static and dynamic compression.

To further deepen our understanding of the material’s temperature-dependent effects, the variation curves of the peak stress and modulus of directional polymethylmethacrylate with temperature under quasi-static and dynamic compression are given in Figure 13. Here, the modulus was obtained using the linear section of the stress–strain curve that was achieved after dynamic equilibrium of the specimen but before yielding. It can clearly be seen that the peak stress and modulus of the material decrease with increasing temperature. The peak stress and modulus under dynamic compression have a certain increase compared with quasi-static compression, which means that this material has strain-rate hardening and strengthening phenomena. As directional polymethylmethacrylate is a viscoelastic material, its failure process is also a relaxation process of the chain segment, so loading rate and temperature have a significant effect on its strength. If the relaxation time of the chain motion in the loading process is consistent with the loading rate, the material may yield before fracture, resulting in forced high elasticity. When the loading rate increases, the motion of the segment cannot keep up with the effect of external force. In order to make the material yield, a larger external force is needed, that is, the yield strength of the material increases; further increasing the loading rate, the material eventually has brittle fractures under higher stress. Therefore, the mechanical behavior of directional polymethylmethacrylate exhibits the strain-rate strengthening effect from quasi-static compression to dynamic compression. In addition, with the increase of temperature, the difference between a quasi-static modulus and dynamic modulus is reduced, which indicates that strain-rate hardening is related to temperature, that is, the strain-rate hardening phenomenon is increasingly weaker with increasing temperature. The difference between dynamic peak stress and static peak stress is not significant, changing with the rise of temperature, which indicates that strain-rate strengthening is less affected by temperature. Under quasi-static loading, the peak stress and modulus of directional polymethylmethacrylate just slightly change when the temperature increases to more than 353 K, which shows that the material has entered a viscous flow state.

### 3.2. Quasi-Static and Dynamic Tensile Response at Different Temperatures

**Figure 14:**
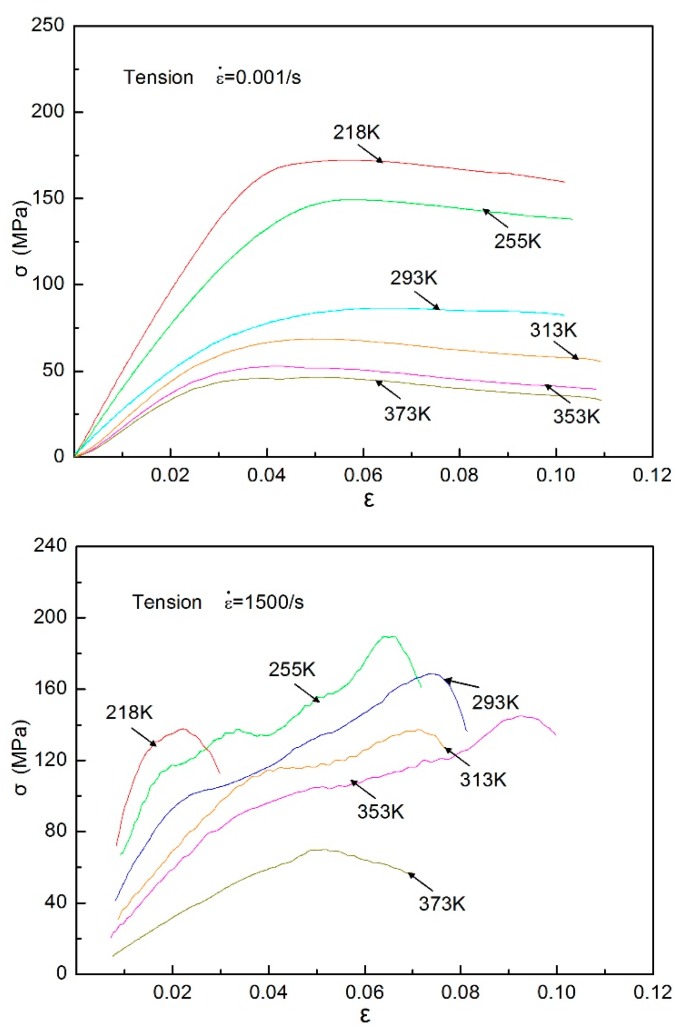
Quasi-static and dynamic tensile stress–strain behavior of directional polymethylmethacrylate at different temperatures.

Quasi-static and dynamic tensile testing for directional polymethylmethacrylate specimens was carried out under different temperatures. The tensile stress–strain behavior of the material was obtained over a wide temperature range of 218 to 373 K, as shown in Figure 14. It can be seen that regardless of quasi-static and dynamic tensile loading conditions, the stress of directional polymethylmethacrylate decreases with the increase of temperature, which shows the effect of temperature softening. For a given temperature, the material has an obvious plastic flow in the process of tensile deformation. Flow characteristics are especially obvious under dynamic tensile loading. In a quasi-static tensile loading regime, the stress–strain curves at temperatures of 353 and 373 K are relatively close to each other, which indicates that the material has entered the deformation state of viscoelasticity. For the high strain rate of 1500/s, directional polymethylmethacrylate at a temperature of 218 K has almost no obvious plastic deformation before failure occurs. This shows that the material undergoes brittle failure in the glassy state. Directional polymethylmethacrylate has large plastic deformation under dynamic tensile loading when the temperature rises from 255 to 353 K, which shows that it is easier for the material to exhibit deformation characteristics of the rubbery state in current conditions. When the test temperature rises to 373 K, the material is damaged under relatively little strain. This is because at higher temperatures it enters a viscous flow state and is more prone to tensile failure.

**Figure 15:**
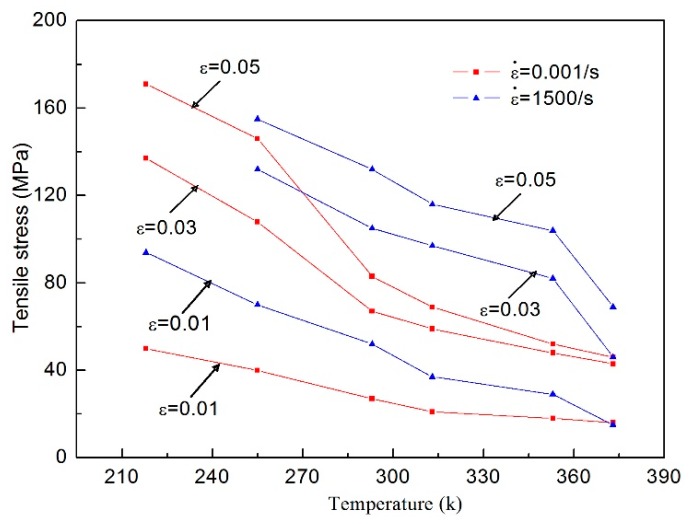
Variation relation of tensile stress with temperature at different strain levels.

**Figure 16:**
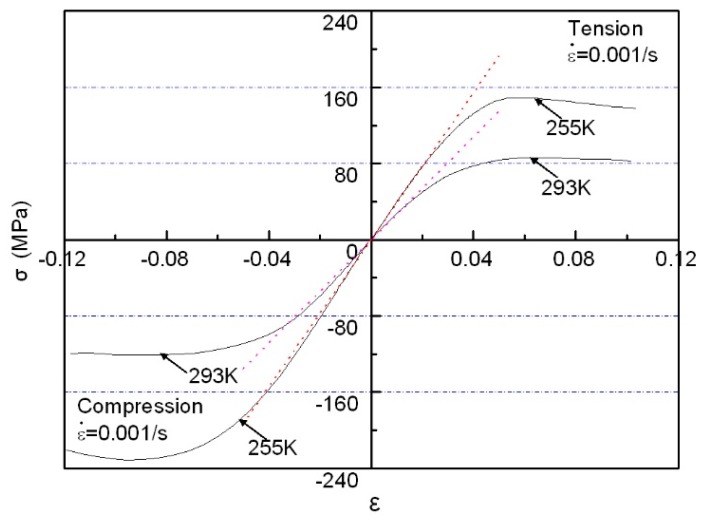
Comparison of quasi-static tensile and compressive curves at two different temperatures.

To further deepen our understanding of the temperature-dependent effects of directional polymethylmethacrylate, Figure 15 gives the relation of tensile stress with changing temperature under different strain levels. It can clearly be seen that under different strain levels, tensile stress decreases with the increase of temperature, and the magnitude of stress that decreases at high strain levels is bigger than at low strain levels. At the same strain and temperature, dynamic tensile stress is much higher than static tensile stress, which also indicates the strain-rate strengthening effect under tensile conditions. The above discussion demonstrates that the stress–strain characteristics of directional polymethylmethacrylate under tension and compression have some differences. To this end, a comparison of quasi-static tensile and compressive curves at two different temperatures is given in Figure 16. It can be found that under the same deformation, the tensile stress of directional polymethylmethacrylate is obviously lower than compressive stress. In the same conditions, tensile stress is almost one-third less than compressive stress. As an amorphous polymer, directional polymethylmethacrylate has mainly three mechanical states: glassy, rubbery, and viscous-flow states. At room temperature and below, its mechanical response is in the glassy state, and only a small deformation occurs under the action of external force. The failure of directional polymethylmethacrylate in the glassy state is mainly caused by brittle fracture. It is well known that for brittle materials, compressive strength is much greater than tensile strength; for plastic materials, compressive strength is approximately equal to tensile strength. With the increase of temperature, the mechanical behavior of directional polymethylmethacrylate gradually changes from the glassy state to the rubbery state. Its failure mode also changes from brittle failure to elastic–plastic failure. Therefore, at the same strain level, the difference between the compressive and tensile stress of the material decreases with the increase of temperature. The above analysis shows that there is asymmetry in the tensile and compressive mechanical properties of directional polymethylmethacrylate.

## 4. Constitutive Model

As it is known, the constitutive modeling of materials is the theoretical basis for numerical calculation and practical engineering design. A constitutive model can also be used to analyze and explain mechanical phenomena observed in various mechanical experiments [21]. Therefore, the accuracy of the constitutive model determines to a large extent whether its calculation results can correctly reflect the mechanical behavior of the materials. The quasi-static and dynamic mechanical behavior of directional polymethylmethacrylate under uniaxial tension and compression deformation is discussed qualitatively in the above section. Here, a visco-hyperelastic constitutive model with thermomechanical coupling effect is introduced to quantitatively describe the mechanical properties of directional polymethylmethacrylate under different loading conditions. The study of Hu et al. [22,23] shows that the general constitutive relation of polymer materials under finite deformation can be decoupled into the following two parts:

(8) $$\sigma =-pI+\Psi \left(E\left(t\right)\right)+\delta \Psi \left(E\left(t\right)|\delta \left(E\left(t-s\right)-E\left(t\right)\right)\right)=-pI+S_{e}+S_{v}\;$$

where *p* is the hydrostatic pressure that controls the volume deformation of the materials, *E*(*t*) and *I* are the strain history and unit tensors, and *S_e_* and *S_v_* are the hyperelastic and viscoelastic deviatoric stress tensors, respectively.

Previous studies have shown that the hyperelastic stress tensor can be expressed as the partial derivative form of the strain-energy function. According to the conclusion of Rivlin et al. [24], the constitutive relation of the hyperelastic stress tensor can be given by:

(9) $$S_{e}=2I_{3}\frac{\partial W}{\partial I_{3}}+2\left[\frac{\partial W}{\partial I_{1}}+I_{1}\frac{\partial W}{\partial I_{2}}\right]B-2\frac{\partial W}{\partial I_{2}}B\cdot B\;$$

where *W* and B are the strain energy function and left Cauchy–Green strain tensor, and *I*_1_, *I*_2_, and *I*_3_ are the three invariants of the Cauchy–Green tensor. At present, there are classical strain-energy functions for hyperelastic deformation, such as the Ogden, Yeoh, and Rivlin functions [24,25,26]. Here, Rivlin’s strain-energy function is selected with the following form:

(10) $$W=\sum_{i+j=1}^{N}C_{ij}{\left(I_{1}-3\right)}^{i}{\left(I_{2}-3\right)}^{j}+\sum_{1}^{N}\frac{1}{D_{i}}{\left(\sqrt{I_{3}}-1\right)}^{2i}\;$$

where *C_ij_* and *D_i_* are the Rivlin coefficients. If the materials are approximately incompressible, then *I*_3_ = 1. Generally speaking, three terms in Rivlin’s strain energy function are sufficient to assure the accuracy of constitutive model:

(11) $$\begin{array}{ll} W & =C_{1}\left(I_{1}-3\right)+C_{2}\left(I_{2}-3\right)+C_{3}\left(I_{1}-3\right)\left(I_{2}-3\right)\\ & =C_{1}\left(\lambda_{1}^{2}+\lambda_{2}^{2}+\lambda_{3}^{2}-3\right)+C_{2}\left(\lambda_{1}^{2}\lambda_{2}^{2}+\lambda_{2}^{2}\lambda_{3}^{2}+\lambda_{1}^{2}\lambda_{3}^{2}\;-3\right)\\ & \;+C_{3}\left(\lambda_{1}^{2}+\lambda_{2}^{2}+\lambda_{3}^{2}-3\right)\left(\lambda_{1}^{2}\lambda_{2}^{2}+\lambda_{2}^{2}\lambda_{3}^{2}+\lambda_{1}^{2}\lambda_{3}^{2}\;-3\right) \end{array}$$

where *C*_1_, *C*_2_, and *C*_3_ are the undetermined constants related to hyperelastic deformation; and *λ*_1_, *λ*_2_, and *λ*_3_ are the principal stretches and *λ*_i_ = 1 + *ε_i_*. Finally, the deviatoric stress tensor for hyperelastic deformation can be given by the substitution of Equation (11) in Equation (9):

(12) $$S_{e}=2\left\{\left[C_{1}+C_{3}\left(I_{2}-3\right)\right]B+\left[C_{2}+C_{3}\left(I_{1}-3\right)\right]\left(I_{1}B-B\cdot B\right)\right\}\;$$

When the material undergoes a viscoelastic response, its mechanical behavior is related to time and deformation rate. That is, the viscoelastic stress state depends not only on the current strain but also on the strain-rate history. So far, some studies have been carried out on the general form of the viscoelastic stress tensor [21,27]. Among them, the viscoelastic functional relationship established by Bernstein and Truesdell can give a good fit to experimental data for various stress states. Following their derivative results [27,28], the deviatoric stress tensor for viscoelastic deformation can be obtained as follows:

(13) $$\begin{array}{ll} S_{v} & =2F\left(t\right)\cdot \int_{-\infty }^{t}\phi \left(I_{1},\;I_{2},\;I_{3}\right)m\left(t-\tau \right)\dot{E}\left(\tau \right)d\tau \cdot F^{T}\left(t\right)\\ & =2\lambda_{i}^{2}\int_{0}^{t}\lambda_{i}\left[C_{4}+C_{5}\left(\sum_{\begin{array}{l} i,j=1\\ i\neq j \end{array}}^{3}\lambda_{i}^{2}\lambda_{j}^{2}-3\right)\right]\exp\left(-\frac{t-\tau }{C_{6}}\right){\dot{\lambda }}_{i}d\tau \end{array}$$

where *C*_4_, *C*_5_, and *C*_6_ are the undetermined constants related to viscoelastic deformation; and *F*(*t*) and *ϕ* are the deformation gradient tensor and damping function, respectively. Under uniaxial tensile and compressive conditions, the principal stretches of polymer materials in the loading direction can be written as *λ*_1_ = *λ* = 1 + *ε*, *λ*_2_ = *λ*_3_ = *λ*^−1/2^ = (1 + *ε*) ^−1/2^. The stress component’s expression of the materials under uniaxial loading can be given by combining Equations (8), (12), and (13), that is:

(14) $$\begin{array}{ll} \sigma_{11} & =-p+2\lambda \left\{C_{1}\lambda +C_{2}+C_{3}\left[\left(\lambda^{2}+2\lambda^{-1}-3\right)+\lambda \left(2\lambda +\lambda^{-2}-3\right)\right]\right\}\\ & \;+2\lambda^{2}\int_{0}^{t}\lambda \left[C_{4}+C_{5}\left(2\lambda +\lambda^{-2}-3\right)\right]\exp\left(-\frac{t-\tau }{C_{6}}\right)\dot{\lambda }d\tau \end{array}$$

(15) $$\begin{array}{ll} \sigma_{22} & =-p+2\lambda^{-2}\left\{C_{1}\lambda +C_{2}+C_{3}\left[\left(\lambda^{2}+2\lambda^{-1}-3\right)+\lambda \left(2\lambda +\lambda^{-2}-3\right)\right]\right\}\\ & -\lambda^{-1}\int_{0}^{t}\lambda^{-2}\left[C_{4}+C_{5}\left(2\lambda +\lambda^{-2}-3\right)\right]\exp\left(-\frac{t-\tau }{C_{6}}\right)\dot{\lambda }d\tau \end{array}$$

The boundary conditions of the specimen under uniaxial loading are considered, then yield *σ*_22_ = 0. Thus, hydrostatic pressure can be obtained as follows:

(16) $$\begin{array}{ll} p & =2\lambda^{-2}\left\{C_{1}\lambda +C_{2}+C_{3}\left[\left(\lambda^{2}+2\lambda^{-1}-3\right)+\lambda \left(2\lambda +\lambda^{-2}-3\right)\right]\right\}\\ & \;-\lambda^{-1}\int_{0}^{t}\lambda^{-2}\left[C_{4}+C_{5}\left(2\lambda +\lambda^{-2}-3\right)\right]\exp\left(-\frac{t-\tau }{C_{6}}\right)\dot{\lambda }d\tau \end{array}$$

The constitutive relation of directional polymethylmethacrylate under one-dimensional loading can be given by substituting Equation (16) into Equation (14).

**Figure 17:**
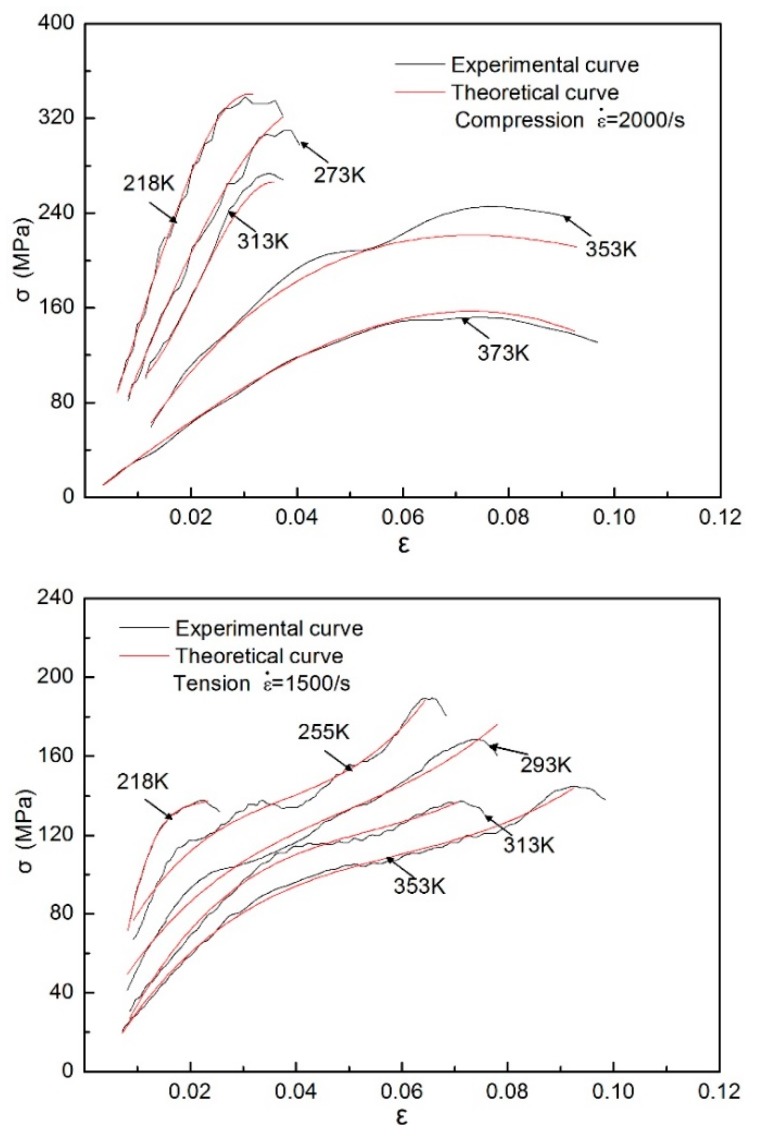
Comparison of experimental results and theoretical predictions of directional polymethylmethacrylate at various temperatures.

**Figure 18:**
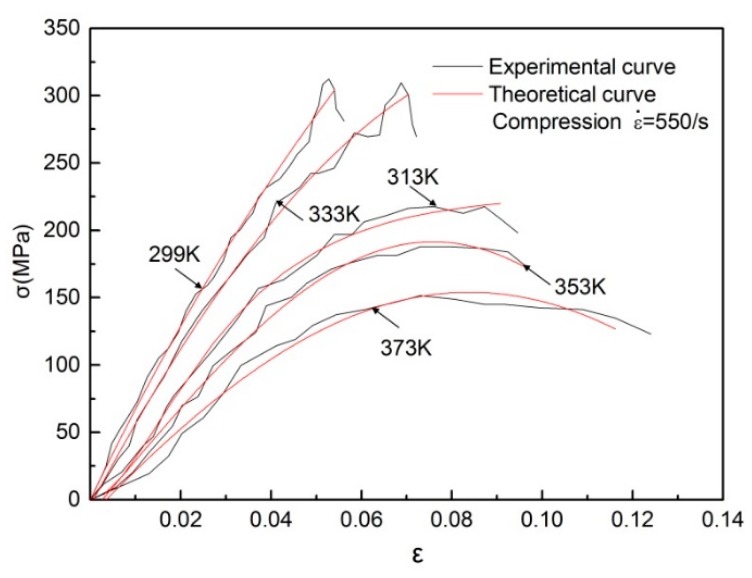
Comparison of model predictions with experimental data on directional polymethylmethacrylate of Suo et al. [30].

The experimental results in Section 3 show that the deformation characteristics of directional polymethylmethacrylate are closely related to temperature, so temperature should also be coupled in the proposed constitutive model. According to the time–temperature equivalence principle of polymer materials and the findings of Maurel-Pantel [29], the temperature-dependent effect of mechanical characteristics can be converted into the effect of equivalent strain rate, that is:

(17) $${\dot{\varepsilon }}_{\mathrm{eq}}=10^{\left[-\frac{A_{1}\left({T-T}_{\mathrm{ref}}\right)}{A_{2}+{T-T}_{\mathrm{ref}}}\right]}\times \dot{\varepsilon }\;$$

where *A*_1_ and *A*_2_ are the material constants, *T* is the test temperature, and $\dot{\varepsilon }$_eq_ is the equivalent strain rate. Here, $\dot{\varepsilon }$ = 1 × 10^−3^/s, and *T*_ref_ = 218 K. Finally, the thermodynamic model can be obtained by substituting Equation (17) into Equation (14). The stress–strain curves of directional polymethylmethacrylate obtained by the experiments are used to evaluate the applicability of the above model, as shown in Figure 17. The value of the model parameters is determined by data fitting with the least-square method, and the values of these parameters are provided in Table 1. Figure 17 shows that the theoretical prediction curves of the constitutive model match well with the experimental data in this paper. To further verify the universality of the newly proposed constitutive model, the theoretical predictions of the model are compared with the available experimental data of Suo et al. [30], as shown in Figure 18. It can be seen that the new model also fits well with the available data in the references, which indicates that the new model is suitable for describing the nonlinear mechanical behavior of directional polymethylmethacrylate under various testing conditions.

## 5. Conclusions

With a newly improved experimental apparatus, a systematic investigation on the tensile and compressive mechanical behavior of directional polymethylmethacrylate was carried out to study the coupled effects of strain rate and temperature. The principles of tension and compression experiments were discussed in detail, and the validity of the experimental data was verified by means of one-dimensional stress wave theory and the DIC technique. The stress–strain characteristics of the material under finite deformation were discussed over a temperature range from 218 to 373 K. The experimental results show that the compressive stress–strain curves of directional polymethylmethacrylate under quasi-static and dynamic loading are temperature-dependent. At a constant strain rate, the stress value for the same level of deformation decreases with the increase of temperature. The peak stress, modulus, and strain-hardening rate of the material showed decreasing trends along with the increase of temperature. The peak stress and modulus of directional polymethylmethacrylate under dynamic compression had a certain increase compared with quasi-static compression, which was manifested as strain-rate hardening and strengthening. Moreover, the strain-rate hardening phenomenon was increasingly weaker with the increase of temperature. The tensile stress–strain characteristics of directional polymethylmethacrylate under quasi-static and dynamic loading had the effect of temperature softening. Under different strain levels, tensile stress decreased along with temperature increase, and the magnitude of decreased stress at high strain levels was bigger than at low strain levels. The tensile stress of directional polymethylmethacrylate in the same loading condition was obviously lower than the compressive stress. As temperature increased, the difference between compressive stress and tensile stress gradually decreased. There was significant asymmetry in the tensile and compressive mechanical properties of directional polymethylmethacrylate. A constitutive model with thermomechanical coupling effects was developed to quantitatively describe the nonlinear mechanical response of the material at different temperatures. By comparing the experimental data with the theoretical results, it was confirmed that the model could well describe the stress–strain behavior of directional polymethylmethacrylate subjected to finite deformation.

## Figures and Tables

**Table 1:** Optimal parameter values determined by the least-square method.

	*C*_1_	*C*_2_	*C*_3_	*C*_4_	*C*_5_	*C*_6_	*A*_1_	*A*_2_
Compression	264	302	−2110	7.5	753	2.6	12.7	63.8
Tension	−52.7	629	1327	−21.4	106	4.1	12.7	63.8
